# A Retained Lens Fragment Lying Dormant for Over 20 Years in the Eye Following Phacoemulsification: A Case Report

**DOI:** 10.7759/cureus.42001

**Published:** 2023-07-17

**Authors:** Chuah Gim Seah, Julieana Muhammed, Lee Annie, Khairuddin Othman

**Affiliations:** 1 Department of Ophthalmology and Visual Science, School of Medical Sciences, Universiti Sains Malaysia, Kubang Kerian, MYS; 2 Department of Ophthalmology, Hospital Sultanah Bahiyah, Alor Setar, MYS

**Keywords:** subacute visual impairment, sulcus intraocular lens implantation, left eye, vitreous liquefaction, dormant retained lens material, posterior vitrectomy surgery, complicated phacoemulsification surgery

## Abstract

A 66-year-old male presented with a three-month history of subacute painless decreased vision in the left eye. His ocular history included complicated phacoemulsification surgery of the left eye more than 20 years prior to the presentation. Slit lamp examination revealed a retained lens nuclear fragment in the superonasal quadrant. Surgical removal of the lens nuclear fragment improved the patient’s condition. The retained lens nuclear fragments were presumably lodged behind the iris during the phacoemulsification surgery and spontaneously displaced downward due to liquefaction of the vitreous body with age. To the best of our knowledge, this case involves one of the longest reported time periods from phacoemulsification surgery to the clinical presentation of retained lens material without causing inflammation. We recommend detailed ocular assessment post complicated phacoemulsification surgery to ensure that no lens materials are retained.

## Introduction

Cataract is the leading cause of blindness, affecting 51% of the population globally [1]. Cataract surgery is one of the most commonly performed surgeries in the world [2]. Charles Kelman introduced the phacoemulsification technique in 1967, which marked a revolutionary change in conventional cataract surgery [1]. In Malaysia, there has been a gradual transformation in the surgical method adopted by ophthalmologists, shifting from conventional extracapsular cataract extraction (ECCE) surgery to the phacoemulsification technique since the early 2000s [1].

While incidences of retained lens materials following phacoemulsification are uncommon, they may result in poor visual outcomes. Retained lens material commonly induces an acute inflammatory reaction in the anterior chamber and is usually detected in the early postoperative period, typically within a few months of the cataract surgery [3]. Delayed postoperative inflammation may occur and manifest as uveitis, exudative retinal detachment, epithelial downgrowth, or masquerade syndromes [4]. There is a scarcity of data in the literature regarding delayed inflammatory reactions induced by retained lens material years after cataract extraction surgery. Tien et al. reported a case of retained lens material causing an inflammatory response approximately 32 years after the cataract extraction surgery [5]. Barnhorst et al. reported the single longest delayed presentation of lens-induced glaucoma due to retained lens material in a patient who presented 65 years after undergoing cataract extraction surgery [6].

There are very few cases of chronic retained lens material that remains in situ without inducing any inflammatory response. We report a case of retained lens material in the retropupillary area that remained dormant for more than 20 years in the left eye following complicated phacoemulsification surgery.

## Case presentation

A 66-year-old male with underlying hypertension, sinus invertus with dextrocardia, and liver cirrhosis presented with subacute painless deterioration of vision in the left eye for three months. The deterioration primarily affected the superonasal quadrant of the visual field. Further history revealed that he had undergone left eye phacoemulsification surgery complicated by a posterior capsule rent and sulcus intraocular lens implantation 20 years ago. Postoperatively, his vision had been good, and he had experienced no visual complaints over the years. Serial follow-up examinations had documented good visual acuity of 6/6 OS (left eye) and no evidence of intraocular inflammation. The patient denied recent trauma or facial injury and had no history of recurrent uveitis or recurrent eye redness with eye pain.

**Figure 1:**
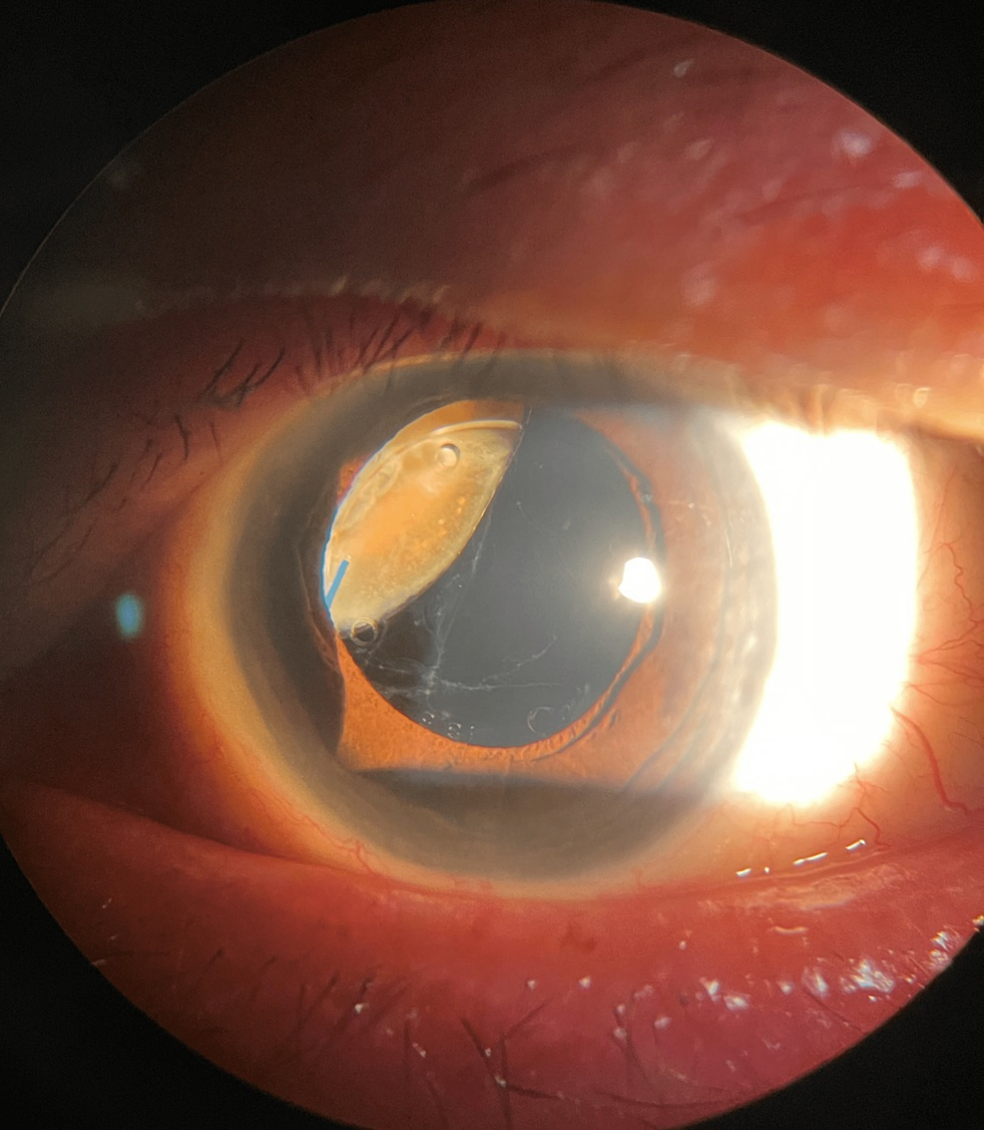
Anterior segment photo of the left eye showing retained large lens nuclear fragment at superonasal quadrant with large posterior capsule rent

During the ocular examination, the left eye exhibited a vision of 6/18, which improved to 6/9 with the pinhole test, while the right eye had a vision of 6/6. Slit lamp examination revealed the presence of a quarter of the lens nuclear fragment at the superonasal quadrant beneath the iris and the implanted intraocular lens (Figure 1). There were no signs of active intraocular inflammation, as indicated by the absence of cells in the anterior chamber, clear cornea without signs of corneal edema, white conjunctiva, and normal intraocular pressure measured at 16 mmHg using Goldmann applanation. Furthermore, a large posterior capsular rent was detected. Fundus examination revealed no abnormalities, and the vitreous was clear.

He subsequently underwent a posterior vitrectomy and lens fragmentation in the left eye, performed by a vitreoretinal surgeon. During the procedure, a large piece of retained lens nuclear material was discovered beneath the iris in the superonasal quadrant. At one month postoperatively, the patient's left eye achieved a best-corrected distance acuity of 6/6. The cornea of the left eye appeared clear, and the anterior chamber was deep and quiet. The posterior chamber intraocular lens remained in place and stable, and the fundus examination revealed no abnormalities.

**Figure 2:**
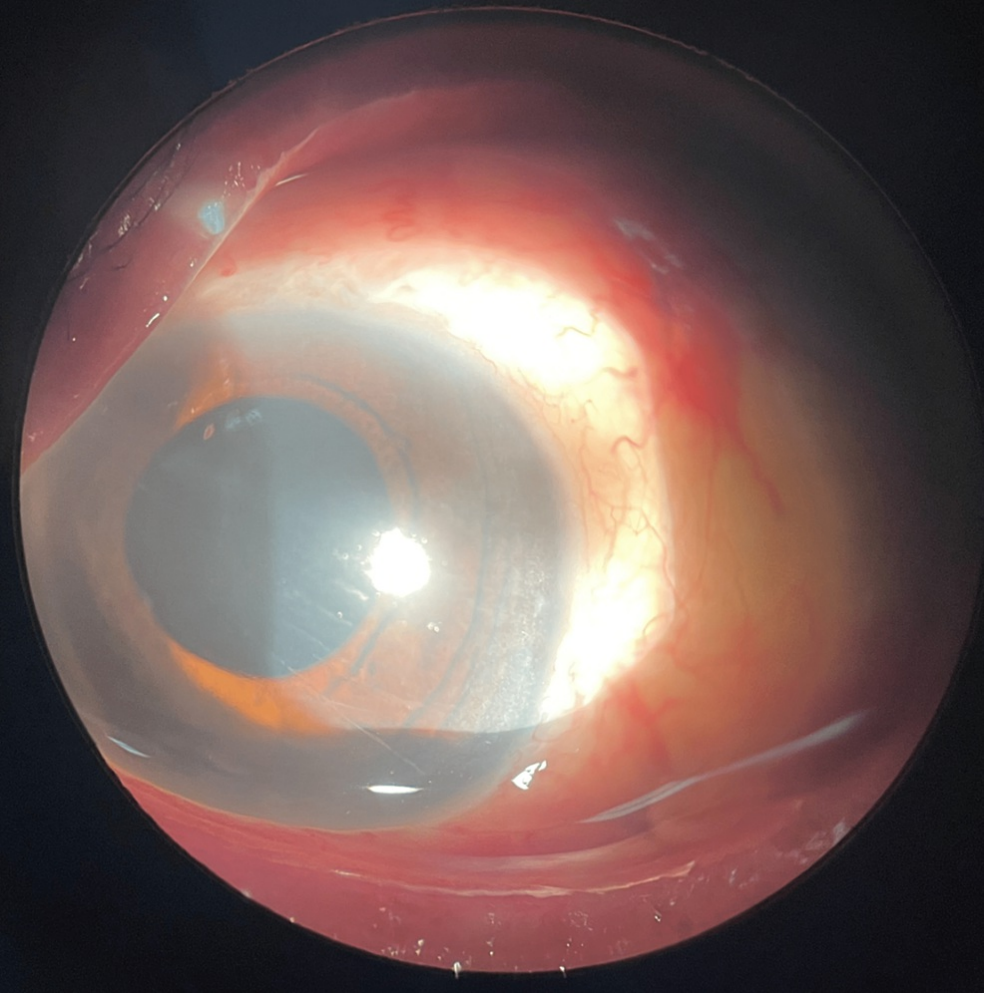
Anterior segment photo of the left eye on day 1 following posterior vitrectomy with lens fragmentation surgery

Figure 2 shows the anterior segment photo of the left eye on postoperative day one.

## Discussion

In this report, we discuss a case of non-inflammatory lens material retained for more than 20 years after complicated phacoemulsification surgery. There is a lack of sufficient data in the literature on delayed ocular manifestations secondary to retained lens material following cataract extraction surgery. Table 1 summarizes the three cases that involve an exceptionally delayed ocular presentation attributed to retained lens materials from cataract extraction surgery.

**Table 1:** Case reports of delayed ocular manifestation secondary to retained lens material post-cataract surgery OD: right eye (oculus dexter)

Case	Patient age/sex	Duration from cataract surgery to presentation	Eye	Presentation	Surgical management	Outcome
Tien et al. [5]	66/male	32 years	OD	Anterior chamber inflammation	Lens fragment aspiration surgery	Anterior chamber inflammation resolved and vision improved
Barnhorst et al. [6]	67/female	65 years	OD	Phacolytic glaucoma	Pars plan vitrectomy	Anterior chamber and vitreous - no inflammation; intraocular pressure normalized without antiglaucoma eyedrops with improved vision
Pandit et al. [3]	79/female	8 years	OD	Cornea edema + iritis	Lens fragment aspiration surgery	Cornea edema and anterior chamber inflammation resolved with improved vision

In contrast, Hui et al. reported a case series of 16 patients who developed corneal edema and iritis due to retained nuclear fragments in the anterior chamber within 182 days of cataract extraction surgery. The mean time for identification of the retained nuclear fragments was 24 days, with two outliers. These two cases required gonioscopy for the detection of the retained nuclear fragments, which were found on days 139 and 182 postoperatively [7]. Oliveira et al. reported two patients who underwent uncomplicated cataract extraction surgery and were incidentally found to have retained nuclear fragments after a bulge in the iris was observed in an otherwise normal anterior chamber. Ultrasound biomicroscopy (UBM) identified the lens fragments at two months and one year post cataract surgery, respectively, in the two patients [8].

An assessment of the cataract surgery trend among ophthalmologists working at hospitals in Malaysia showed an increase in the change of surgical technique from ECCE to phacoemulsification, with more than 50% of surgeries being performed using phacoemulsification in 2005 [1]. The transition from ECCE to the newly introduced phacoemulsification technique among ophthalmologists in Malaysia in the early 2000s increased the risks of intraoperative complications. Surgeons are still on a learning curve with the new technique and equipment, which contributes to the increased risks of complications following cataract surgery. The decline in incidence over time may be attributed to advancements in phacoemulsification technologies and the utilization of more efficient phacodynamics. Dislocation of lens materials into the vitreous cavity typically occurs following the rupture of the posterior capsule or zonular dehiscence.

Our patient had undergone a complicated phacoemulsification surgery, resulting in the retention of lens nuclear fragments in the posterior segments. Surprisingly, there had been no signs of inflammation during the 20 years before his current presentation. This case involves one of the longest delayed presentations of retained lens nuclear fragments without causing ocular inflammation after cataract surgery. The exact pathophysiology of retained lens fragments failing to cause inflammation remains unclear. It has been hypothesized in the (limited) literature that the lens nuclear fragments could be lodged within the iris crypts or beneath the iris at the posterior segment. Alternatively, the nuclear fragments may get sequestered in the posterior capsule, masking the inflammatory event after the surgery. Throughout this prolonged duration, the lens fragment may not induce inflammation in the anterior chamber or result in cystoid macular edema [5].

Despite the fact that the patient had no history of recent trauma or injury, the downward migration of the retained lens nuclear fragment could be due to the normal process of vitreous liquefaction. The process of vitreous liquefaction may commence as early as the age of four years, and by the age of 90 years, the vitreous humor may be more than 50% liquefied. During the senescence, the vitreous volume will decrease and the vitreous body will collapse (syneresis), with the fibers thickening, becoming tortuous, and getting surrounded by liquid vitreous [9].

## Conclusions

While incidences of lens material getting retained are rare, the risk is higher following complicated phacoemulsification surgery. Although acute and delayed inflammatory responses are the common sequelae, they may remain dormant for years without inducing inflammatory reactions. We recommend conducting a comprehensive ocular examination in all cases of complicated cataract surgeries in order to detect any missed residual lens material. Surgical removal of the retained lens material led to good visual outcomes in our patient.
